# Downwelling longwave radiation and sensible heat flux observations are critical for surface temperature and emissivity estimation from flux tower data

**DOI:** 10.1038/s41598-022-12304-3

**Published:** 2022-05-21

**Authors:** Gitanjali Thakur, Stanislaus J. Schymanski, Kaniska Mallick, Ivonne Trebs, Mauro Sulis

**Affiliations:** Environmental Sensing and Modelling Unit (ENVISION), Environmental Research and Innovation Department (ERIN), Luxembourg Institute of Science and Technology (LIST), Belvaux, Luxembourg

**Keywords:** Atmospheric science, Hydrology, Biophysics, Climate sciences, Environmental sciences

## Abstract

Land surface temperature (LST) is a preeminent state variable that controls the energy and water exchange between the Earth’s surface and the atmosphere. At the landscape-scale, LST is derived from thermal infrared radiance measured using space-borne radiometers. In contrast, plot-scale LST estimation at flux tower sites is commonly based on the inversion of upwelling longwave radiation captured by tower-mounted radiometers, whereas the role of the downwelling longwave radiation component is often ignored. We found that neglecting the reflected downwelling longwave radiation leads not only to substantial bias in plot-scale LST estimation, but also have important implications for the estimation of surface emissivity on which LST is co-dependent. The present study proposes a novel method for simultaneous estimation of LST and emissivity at the plot-scale and addresses in detail the consequences of omitting down-welling longwave radiation as frequently done in the literature. Our analysis uses ten eddy covariance sites with different land cover types and found that the LST values obtained using both upwelling and downwelling longwave radiation components are 0.5–1.5 K lower than estimates using only upwelling longwave radiation. Furthermore, the proposed method helps identify inconsistencies between plot-scale radiometric and aerodynamic measurements, likely due to footprint mismatch between measurement approaches. We also found that such inconsistencies can be removed by slight corrections to the upwelling longwave component and subsequent energy balance closure, resulting in realistic estimates of surface emissivity and consistent relationships between energy fluxes and surface-air temperature differences. The correspondence between plot-scale LST and landscape-scale LST depends on site-specific characteristics, such as canopy density, sensor locations and viewing angles. Here we also quantify the uncertainty in plot-scale LST estimates due to uncertainty in tower-based measurements using the different methods. The results of this work have significant implications for the combined use of aerodynamic and radiometric measurements to understand the interactions and feedbacks between LST and surface-atmosphere exchange processes.

## Introduction

The effects of global change are reflected in land surface temperature (LST) anomalies and their interannual variability^1^. LST controls the magnitude and variability of the surface energy balance (SEB) components and simultaneously gets modulated by the SEB partitioning^2,3^. LST contains imprints of surface moisture and is extremely sensitive to evaporative cooling, which makes it a preeminent variable for studying evaporation and surface-atmosphere exchange^4–6^. It directly affects the amount of emitted longwave radiation and influences the saturation vapor pressure at the surface that drives latent heat flux. Thus, the ecohydrological functioning and carbon-water coupling are largely controlled by the surface temperature of the soil-vegetation system^7^. The availability of an extensive network of eddy covariance measurements (FLUXNET) allows us to understand the interactions and feedbacks between the surface-atmosphere exchange processes such as evaporation, transpiration, and its control by the atmosphere and vegetation at the diurnal time scale. However, the unavailability of direct LST measurements at the same scale hinders a detailed understanding of the interactions and feedbacks between LST and surface-atmosphere exchange processes, which is of utmost importance to the climate modeling community^8^.

Inversion of the longwave radiation in FLUXNET data to obtain LST has gained popularity in recent years. LST estimation depends on the emissivity of the underlying surface^9^, which is not available as routine measurement. Therefore, estimating in-situ LST is not straightforward due to the involvement of two unknowns (LST and emissivity) inside one measurement variable (upwelling longwave radiation). To circumvent this challenge, we conducted simultaneous retrievals of LST and emissivity by exploiting the longwave radiation components in conjunction with associated SEB flux measurements^10,11^.

The SEB components can be sub-divided into radiative components (often lumped in net radiation, $R_{net}$) and thermodynamic components, including sensible, latent and ground heat flux (*H*, *LE*, *G* respectively):

1 $$\begin{aligned} R_{net} = H + LE + G \end{aligned}$$

The instantaneous value of LST is the result of interplay between the net radiation at the surface, ground heat flux (*G*), sensible heat flux (*H*) and latent heat flux (*LE*)^12^. Thus, LST can also be used for the estimation of *H*^13^ and *LE*^14^ between the surface and the atmosphere. LST provides the lower-boundary condition in SEB models for diagnostic estimates of LE and is highly relevant for drought monitoring^2,5,15^. As the surface-to-air temperature difference drives the exchange of sensible heat between surface and atmosphere, all components of Eq. (1) depend on the LST.

Net radiation ($R_{net}$) can be sub-divided into downwelling and upwelling components^16^ as shown below:

2 $$\begin{aligned} R_{net} = R_{sdwn} + R_{ldwn} - R_{sref} - R_{lref} - R_{lem} \end{aligned}$$

Only a fraction of solar top-of-the-atmosphere radiation reaches the Earth’s surface, as some is reflected back to space by clouds, some is absorbed by the atmosphere and emitted later as longwave radiation. Reflected shortwave in Eq. (2) is expressed as $R_{sref} = \alpha R_{sdown}$, while reflected longwave is represented as $R_{lref} = \alpha R_{ldown}$, where $\alpha$ is the surface albedo. The emitted longwave radiation as a function of surface temperature ($T_s$) and surface emissivity ($\varepsilon$) is given by the Stefan-Boltzmann (SB) equation^17^

3 $$\begin{aligned} R_{lem}= \varepsilon \sigma T_{s}^{4} \end{aligned}$$

where $\sigma$ ($\hbox {W m}^{-2}\,\hbox {K}^{-4}$) is the SB constant, $\varepsilon$ is the surface emissivity ranging between 0 and 1, and $T_{s}$ (K) is the LST. For a land surface, emissivity depends on soil type, vegetation cover, soil moisture, soil chemistry, roughness, spectral wavelength, temperature and view angle^18^.

The emitted and downwelling longwave radiance are measured at given angle within its instantaneous field of view (fov) by a downward facing sensor relatively close to the surface (a few meters for an eddy covariance tower). The radiation received by a pyrgeometer or infrared sensor is a combination of the radiation emitted ($R_{lem}$) and reflected ($R_{lref}$) by the surfaces in its fov as shown in Eq. (4):

4 $$\begin{aligned} R_{lup} = R_{lem} + R_{lref} \end{aligned}$$

Substitution of Eq. (3) into Eq. (4) and replacing $\alpha$ as $1 - \varepsilon$, $R_{lup}$ becomes a function of emissivity, surface temperature and downwelling longwave radiation:

5 $$\begin{aligned} R_{lup}= \varepsilon \sigma T_{s}^{4} + (1- \varepsilon )R_{ldwn} \end{aligned}$$

Equation (5) is then solved for LST as a function of measured longwave and known surface emissivity:

6 $$\begin{aligned} T_{s} = \root 4 \of {\frac{R_{ldwn}}{\sigma } - \frac{R_{ldwn}}{\varepsilon \sigma } + \frac{R_{lup}}{\varepsilon \sigma }} \end{aligned}$$

In order to invert LST as shown in Eq. (6), $\varepsilon$ values are required. However, radiometers at eddy covariance sites (ECS) do not measure spectral bands separately to deduce emissivity directly. Therefore, we will deduce site specific $\varepsilon$ from observations of air temperature ($T_a$), measured longwave ($R_{ldwn}$, $R_{lup}$) and sensible heat flux (*H*)^19^. In analogy to Ohm’s law, the linear relationship between *H* and $\Delta T$ can be expressed mathematically as:

7 $$\begin{aligned} H= m(T_{s} - T_{a}) \end{aligned}$$

where *m* ($\hbox {m s}^{-1}$) is a proportionality constant (defined as $m=\rho C_{p}/r_{a}$ and broadly referred to as heat transfer coefficient) and depends on surface characteristics and micro-meteorology^17^, $T_{a}$ (K) is the temperature of the air measured at a reference height above the surface, $C_{p}$ ($\hbox {J kg}^{-1}$ $\hbox {K}^{-1}$) is the specific heat capacity of air, $\rho$ ($\hbox {kg m}^{-3}$) is the air-density, and $r_{a}$ ($\hbox {s m}^{-1}$) is the total resistance to heat transport from surface to the atmosphere. It is evident from Eq. (7) that for $T_{s} - T_{a} = 0$, *H* will be zero. This boundary condition and the linear relationship between *H* and $\Delta T$ is used to estimate $\varepsilon$^10,19^. Another approach for plot-scale $\varepsilon$ estimation filters the data where *H* is close to zero, substitutes $T_{s}$ in Eq. (5) by $T_{a}$ and solves for $\varepsilon$^11^.

However, due to surface heterogeneity, sparse canopies are prone to footprint mismatch between the aerodynamic (flux tower) footprint and radiometric (hemispherical) footprint^20–22^, where the aerodynamic footprint represents the area contributing to measured sensible heat flux, while the radiometric footprint is dominated by the surface below the sensor at a nadir viewing angle, contributing to the measured longwave radiation (used for $T_{s}$ estimation). This can result in a different boundary condition i.e. at $\Delta T =0$, $H \not = 0$ as expressed in Eq. (8):

8 $$\begin{aligned} H= m(T_{s} - T_{a}) + c \end{aligned}$$

where *H* is representative of the sensible heat flux from the eddy covariance tower footprint, $T_{s}$ is representative of all the radiating surfaces in the radiometric sensor’s view, and *c* is interpreted as the *H* from surfaces in the aerodynamic footprint that are not seen by the radiometer.

Plot-scale estimation of $\varepsilon$ and LST using observed *H*, $T_{a}$, $R_{lup}$ and $R_{ldwn}$ as described above and in the Methods section, may be prone to substantial uncertainty. It is unclear how uncertainties in observed fluxes propagate into the uncertainty of estimated LST and $\varepsilon$. By design, infrared thermal (IRT) sensors only measure upwelling infrared radiance and therefore cannot explicitly account for the amount of reflected downwelling infrared radiation in the signal. For a long time, downwelling longwave ($R_{ldwn}$) was not routinely observed at ECS^23^ and was also considered to be the most poorly quantified component of the radiation budget^24^. Therefore, the second term in Eq. (5) is commonly omitted, arguing that $\varepsilon \approx 1$, and hence, Eq. (5) is simplified to Eq. (3)^25^:

9 $$\begin{aligned} R_{lup} \approx \varepsilon \sigma T_{s}^{4} \end{aligned}$$

Equation (9) can be solved for $T_s$ to yield what we will term the “short equation” (seq) for $T_s$:

10 $$\begin{aligned} T_{s} \approx \root 4 \of {\frac{R_{lup}}{\varepsilon \sigma }} \end{aligned}$$

Note that the above derivation is actually flawed, as the second term of Eq. (5) was omitted arguing that $\varepsilon \approx 1$, and yet $\varepsilon$ was retained in the first part of the equation. Nevertheless, even with the availability of downwelling longwave measurements^26^, the use of Eq. (9) is still a common practice^9,25^. This gives rise to the question if the short equation [Eq. (10)] is adequate to estimate LST from ground-based measurements. In the remainder of this paper, we will refer to LST obtained using the long equation [Eq. (6)] as $T_{leq}$ and to LST obtained using the short equation [Eq. (10)] as $T_{seq}$.

To better understand and improve approaches of plot-scale LST estimation, the present study addresses the following research questions:

1. Can we obtain an adequate estimate of plot-scale LST while neglecting the reflected downwelling longwave radiation?
2. Does the estimation of plot-scale $\varepsilon$ based on observed sensible heat flux (*H*) have an advantage over satellite-derived $\varepsilon$ for plot-scale LST estimation?
3. How much uncertainty is introduced in plot-scale LST and $\varepsilon$ due to uncertainty in measured EC fluxes?

To answer these questions, we analysed data for ten eddy covariance sites in different biomes and climates (see Table 2). Plot-scale broadband monthly emissivity ($\varepsilon _{plot}$) was derived using observed *H* and estimated $\Delta T$ as proposed by Holmes et al.^10^. Plot-scale LST was estimated using either Eqs. (6) or (10), and either $\varepsilon _{plot}$ or landscape-scale emissivity ($\varepsilon _{MODIS}$). Estimated LST was compared with MODIS LST (TERRA satellite-sensed) for the times of satellite overpass. Uncertainty in $\varepsilon _{plot}$ and LST due to uncertainty in observed fluxes was calculated using SOBOL-based uncertainty analysis (SAlib)^27^. See the Methods section for more details.

## Results

### Plot-scale $\varepsilon$ using long and short equation

**Figure 1:**
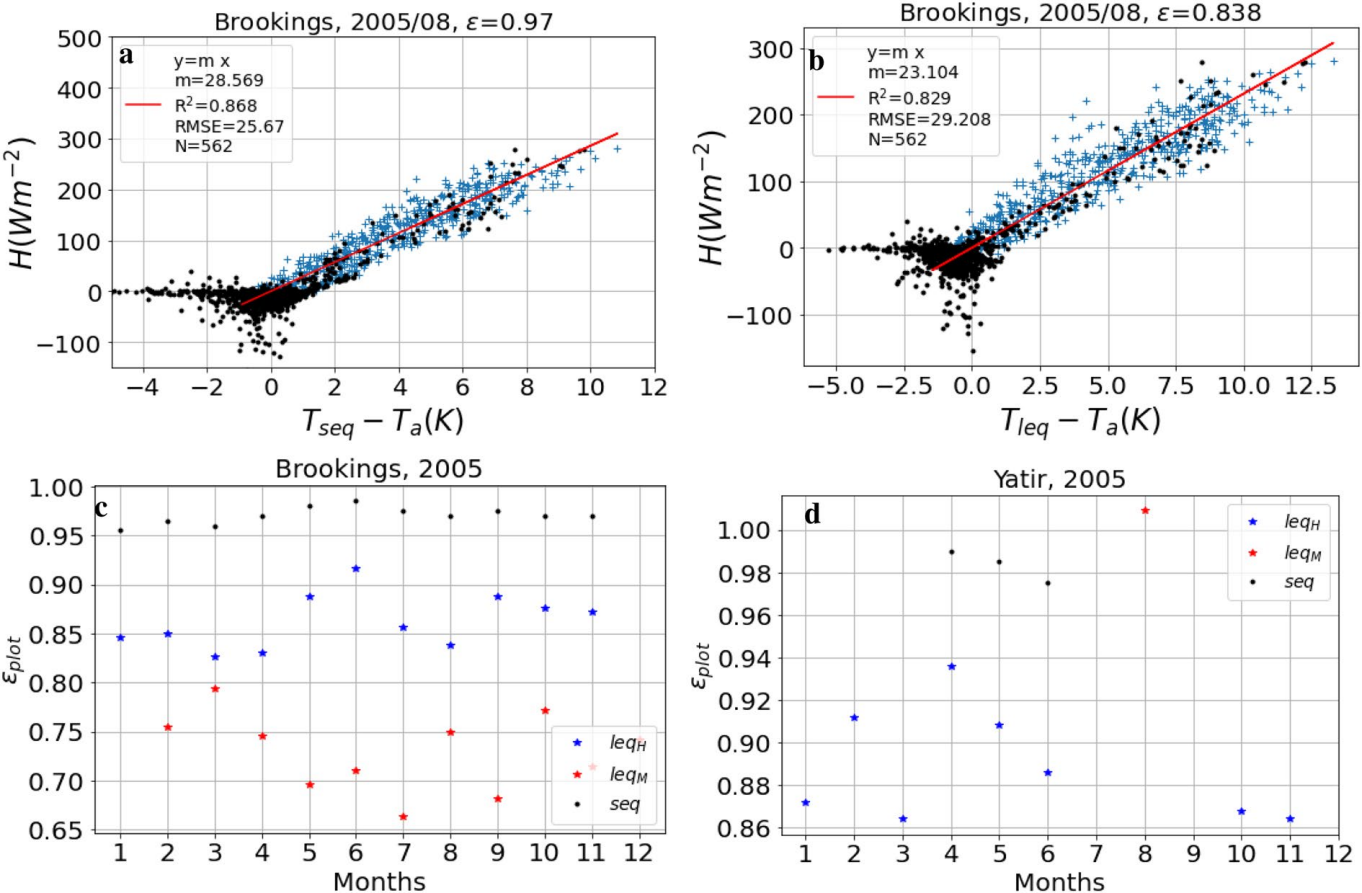
Reproduction of analysis presented in Figs. 2a, 3C, and Q in Holmes et al.^10^. (**a**) Sensible heat (*H*) vs. $\Delta T = T_{seq} - T_{a}$ based on the short equation [$T_{seq}$, Eq. (10)]; (**b**) *H* vs. $\Delta T$ based on the long equation ($T_{leq}$, Eq. 6). Both show data for August 2005 at Brookings. Blue crosses represent data points satisfying the filtering criteria, while black dots represent points not considered in the analysis. N is the number of blue crosses used for regression (red line), m is the slope of regression, RMSE is the root mean square error and R$^{2}$ is the square of the coefficient of determination. The fitted $\varepsilon$ value is reported in the title. (**c**) Optimised $\varepsilon$ values at Brookings obtained for the months where $\hbox {R}^{2} > 0.5$ using the short equation (Eq. 9, black dots) and long equation (Eq. 6, blue stars), and $\varepsilon$ obtained using the approach of Maes et al.^11^ (red stars). (**d**) Same as (**c**), but for Yatir Forest, see Table 2 for site descriptions.

**Figure 2:**
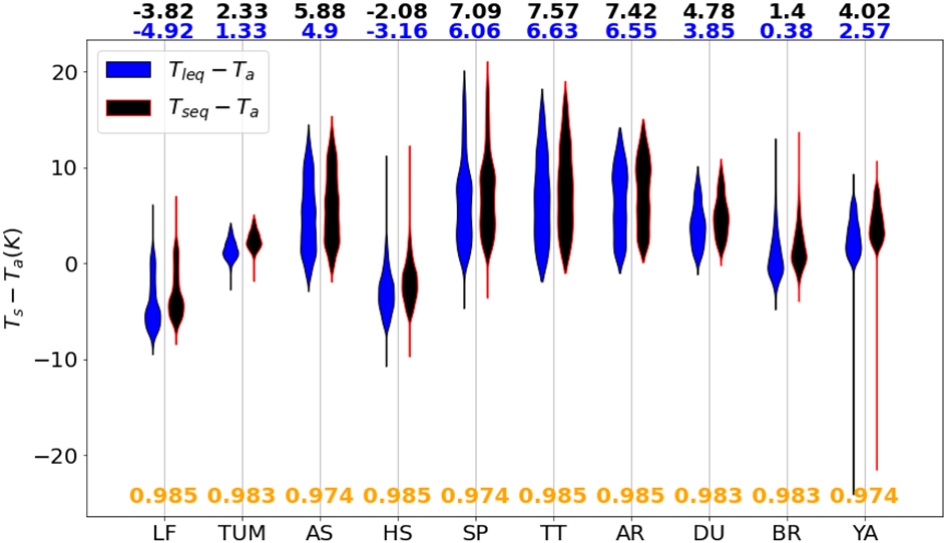
Yearly distributions of half-hourly surface-to-air temperature differences ($\Delta T = T_s - T_a$) for a representative year at each site. LST was calculated using the short equation [Eq. (10)] or long equation [Eq. (6)] with landscape-scale emissivity ($\varepsilon _{MODIS}$). The median values of $\Delta T$ are shown at the top of the plot and the $\varepsilon _{MODIS}$ values used for the $T_{s}$ retrieval are shown at the bottom in orange. See Table 2 for site abbreviations. The shapes of the violin represent the distributions of $\Delta T$ values.

**Figure 3:**
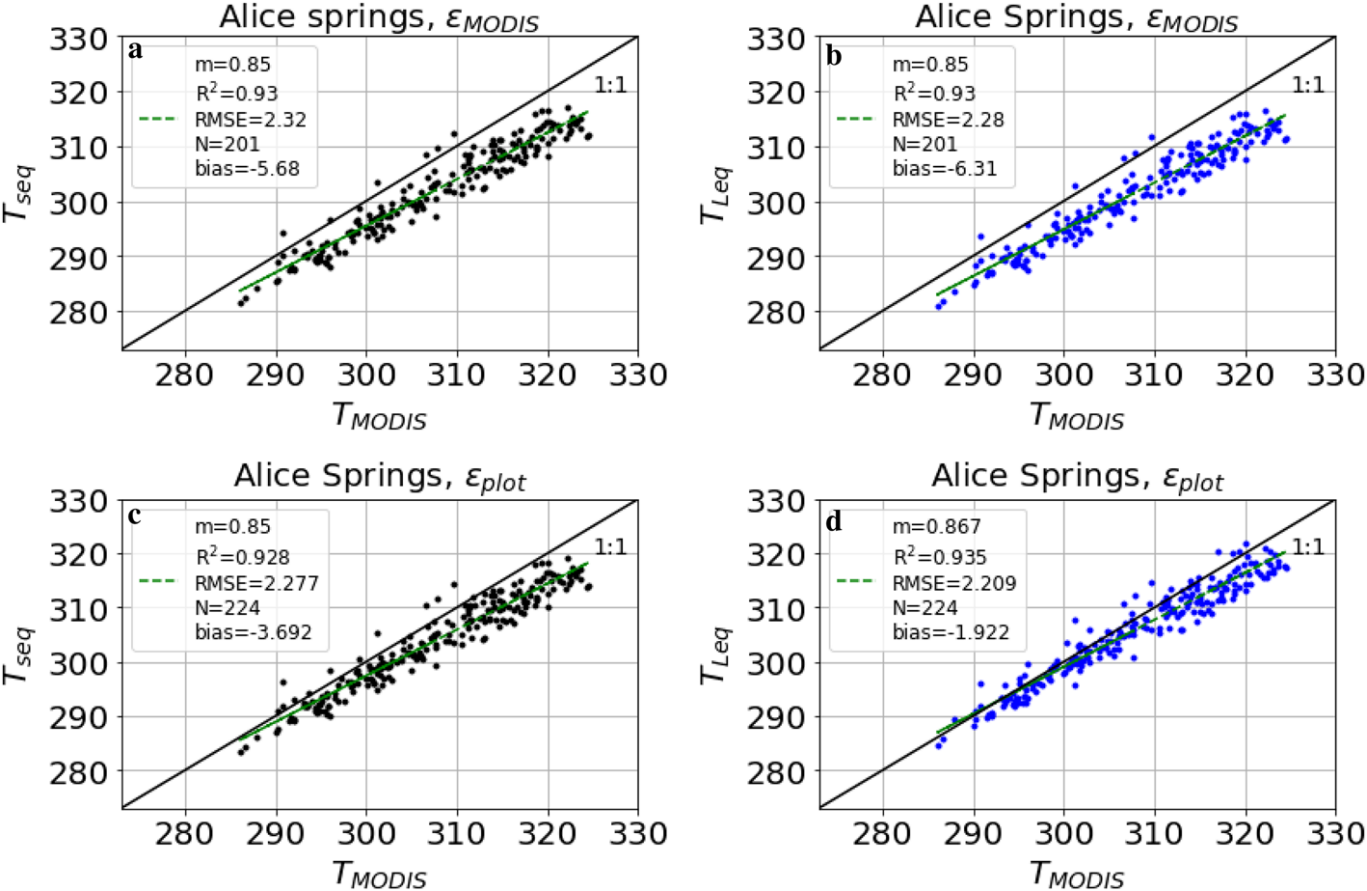
Landscape-scale LST ($T_{MODIS}$ derived from MOD11A1) vs. plot-scale LST at Alice Springs for 2016–2018. (**a**) $T_{seq}$ based on short equation (Eq. 10) and satellite-derived (MODIS) broadband emissivity; (**b**) Same as (**a**), but $T_{leq}$ based on long equation (Eq. 6); (**c**) $T_{seq}$ based on short equation (Eq. 10) and monthly plot-scale emissivity; (**d**) Same as (**c**), but $T_{leq}$ based on long equation (Eq. 6). Bias is mean $T_{seq} - T_{MODIS}$, N is the number of daily overpasses of MODIS between 2016 and 2018, c is the intercept, m the slope, RMSE is the root mean square error and $R^{2}$ is the coefficient of determination. At each site, LST was estimated using both the short equation ($T_{seq}$, Eq. 10) and the long equation ($T_{leq}$, Eq. 6). In a first step, we used satellite-derived landscape-scale broadband emissivity from MODIS ($\varepsilon _{MODIS}$, Eq. 12) for estimating plot-scale LST from tower-based longwave measurements, and compared these with landscape-scale LST extracted from MODIS LST dataset ($T_{MODIS}$).

**Figure 4:**
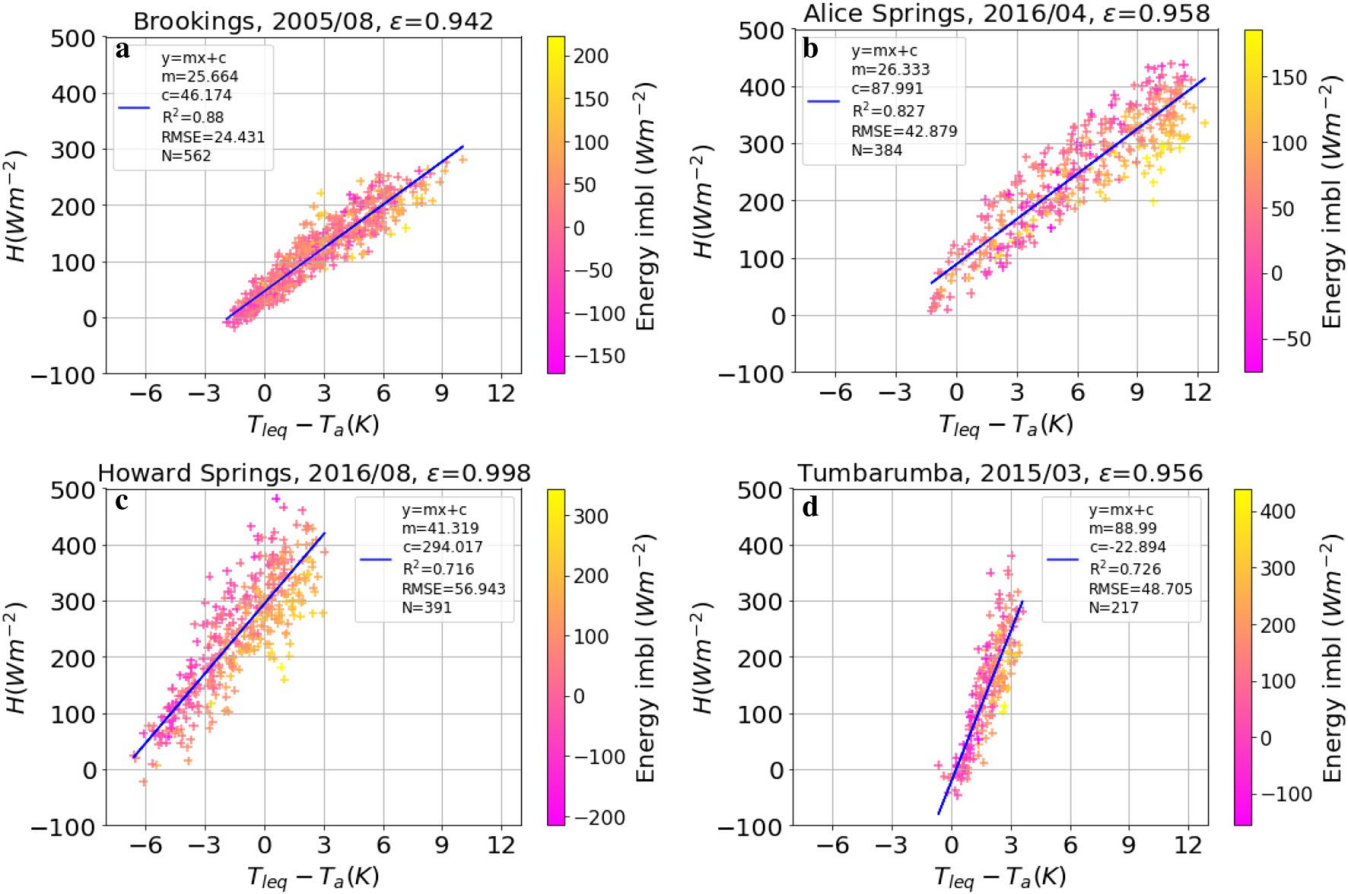
Sensible heat flux as a function of surface-to-air temperature difference based on Eq. (8) ($H = m (T_{s} - T_{a}) + c$). $\varepsilon$ was fitted to minimise RMSE of a robust linear regression. The title of the plot contains site, year, month and the fitted $\varepsilon$-value. The legend correspond to Fig. 1. The colour code indicates the degree of energy imbalance of each data point (i.e. $R_{net} - H - LE - G$). The panels (**a**) to (**d**) refer to the same analysis at different sites, as indicated in the title of each panel.

Following the method proposed by Holmes et al.^10,19^, plot-scale monthly $\varepsilon$ was estimated at the study sites by fitting $\varepsilon$ to minimise the root mean square error (RMSE) of the regression between *H* and $T_s - T_a$ (see SI Figure 3). In Fig. 1a, c, and d, we used the original data and reproduced Figs. 2a, 3C, and 3Q from Holmes et al.^10^ to validate our interpretation of their approach using the short equation (Eq. (10)). We noted only marginal differences between the two results based on the short equation, which are likely due to different fitting algorithms. The replication of the $H (\Delta T)$ plot using the long equation [Eq. (6)] with the same data is given in Fig. 1b and the monthly $\varepsilon$ values are shown in Fig. 1c, d, indicated by blue stars. The retrieved LST values were slightly higher when using Eq. (6) (compare a and b in Fig. 1). The use of the long equation [Eq. (6)] resulted in substantially (10%) lower values of $\varepsilon$ as compared to the values estimated by Holmes et al.^10^ for the common study sites (Brookings, Fig. 1c and Yatir, Fig. 1d). The reduction in $\varepsilon$ can be attributed to the sensitivity of the two equations to the emissivity. As shown in the SI (Fig. 4), the $T_{s}$ estimation using the short equation is more sensitive to $\varepsilon$ than for the long equation, thus even a small reduction in $\varepsilon$ can lead to a large increase in the $T_{s}$ (to minimise RMSE).

Another approach for plot-scale $\varepsilon$ estimation (Maes et al.^11^) in combination with Eq. 6) resulted in even lower $\varepsilon$ values for Brookings, as shown in Fig. 1c (red stars), whereas at Yatir, this approach gave an $\varepsilon$ value higher than 1 (red star in Fig. 1d). Note that the long equation also yielded an acceptable $H(\Delta T)$ relationship for more months at Yatir Forest (blue stars) than the short equation (black dots), as shown in Fig. 1d. The pattern of lower $\varepsilon$ and higher LST using the long equation compared to the short equation was confirmed for all the ten sites used in the present study (SI Table 2).

### Landscape-scale vs plot-scale estimates of $\varepsilon$ and LST

At each site, LST was estimated using both the short equation ($T_{seq}$, Eq. 10) and the long equation ($T_{leq}$, Eq. 6). In the first step, tower-based longwave radiation and landscape-scale broadband $\varepsilon$ from MODIS spectral $\varepsilon$ ($\varepsilon _{MODIS}$, Eq. 12) was used. The yearly daytime surface-to-air temperature difference for each study site is estimated and shown in Fig. 2. At all sites, Eq. (10) resulted in higher day-time plot-scale $T_{s}$ estimates as compared to Eq. (6), when using $\varepsilon _{MODIS}$, with the medians of surface-to-air temperature differences ($\Delta T$) differing by 0.8–1.5 K (Fig. 2). The difference in $\Delta T$ using the two equations is highest at the water limited sites, e.g. AS and YA. Note that for two sites (LF and HS), the median values of daytime $\Delta T$ are negative. Comparison of plot-scale LST estimated using $\varepsilon _{MODIS}$ at satellite overpass time with landscape-scale LST ($T_{MODIS}$) revealed strong correlations at most of study sites but systematically lower plot-scale LST (Fig. 3a, b). Use of $\varepsilon _{plot}$ for LST estimation ($T_{seq}$ and $T_{leq}$) resulted in substantial reduction of the bias as shown in Fig. 3c, d. This trend in bias reduction was similar at other sites (SI Table 2 for details). The minimum bias is found at TUM, a closed canopy (eucalyptus forest) and the highest bias was obtained at LF and HS, heterogeneous ecosystems with sparse canopies (woodland savanna). However, for some sites, weak correlation between satellite-derived and local LST estimates were also evident (at DU, $R^2$ was reduced from 0.8 to 0.4, see SI Table 2). The low correlation between MODIS LST and plot-scale LST can be due to various reasons, such as differences in sensor types, viewing angles and distance between the sensors and sources, e.g. requiring atmospheric correction for satellite-based sensors. Also, using plot-scale $\varepsilon$ for LST estimation resulted in positive $T_{s} - T_{a}$ at LF and HS as shown in SI Figure 1 in comparison to Fig. 2.

### Plot-scale $\varepsilon$ estimation using long equation with intercept

**Figure 5:**
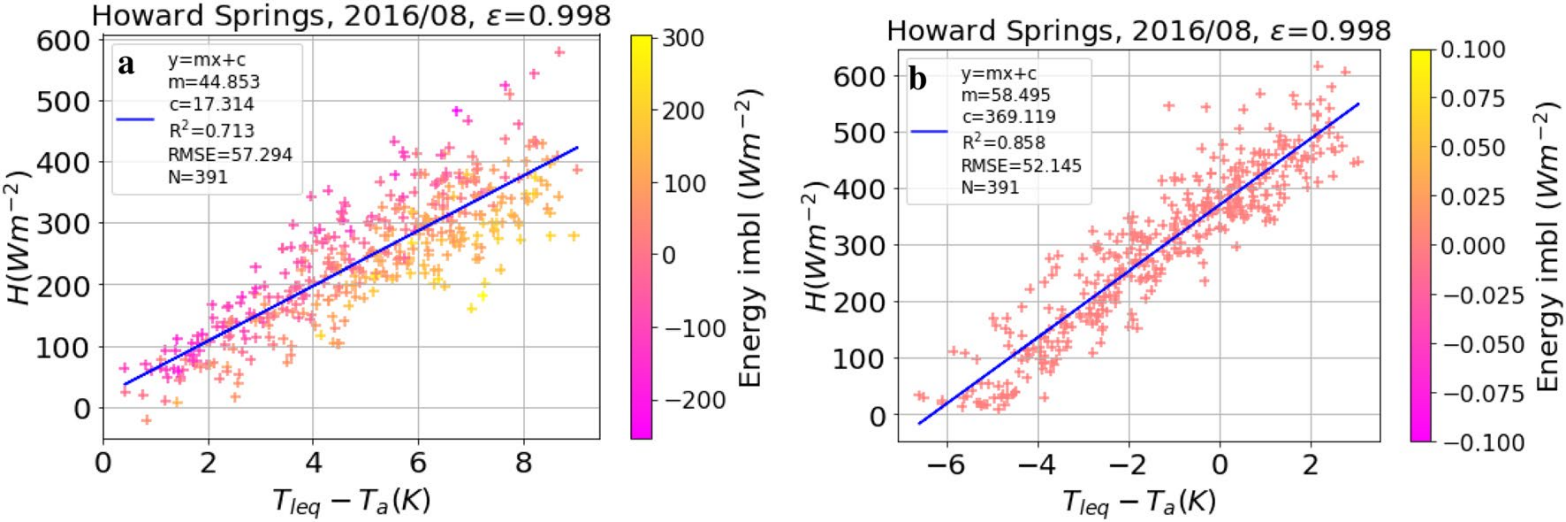
Sensible heat flux as a function of surface-to-air temperature difference based on Eq. (8) ($H = m (T_{s} - T_{a}) + c$). Same analysis and legends as in Fig. 4c), but (**a**) after adding 40 $W m^{-2}$ to measured $R_{lup}$, and (**b**) after closing the energy imbalance using a Bowen ratio closure scheme.

In order to account for the possibility of bias between radiometric and aerodynamic measurements (e.g. due to footprint mismatch of measuring devices or instrument bias), we also fitted Eq. (8), i.e. a relationship allowing for an intercept in the linear fit between *H* and $\Delta T$ (instead of forcing it through zero as in Fig. 1) for plot-scale $\varepsilon$ estimation. As shown in Fig. 4, the plot-scale $\varepsilon$ values resulting from this approach ($H=m \Delta T + c$) were substantially closer to the landscape-scale $\varepsilon$ values compared with the approach without intercept ($H=m \Delta T$), as shown in Table 1. However, comparison of the resulting plot-scale LST with landscape-scale LST values revealed an increase in bias at most sites compared to the LST obtained using $\varepsilon _{plot}$ without an intercept (Table 1). The median values of the resulting intercept ranged from − 24 to $+$ 258 $\hbox {W m}^{-2}$, with the highest intercept values at Howard Springs (amounting to 70% of the maximum observed *H* at this site). The minimum value of intercept was obtained at Tumbarumba (5% of the maximum observed *H*). Note, that if we assumed just a slight under-estimation of upwelling longwave radiation by 40 $\hbox {W m}^{-2}$ at Howard Springs (ca. 8% of observed $R_{lup}$), the intercept was reduced from 294 (Fig. 4c) to 17 $\hbox {W m}^{-2}$ (Fig. 5a) without change in other regression paramaters (m, RMSE, $R^{2}$). In this study, we did not apply any energy balance closure scheme, as a Bowen ratio closure, although resulting in higher $R^{2}$ values at HS, also led to even greater intercept (c) (Fig. 5b). Interestingly, adding 40 $\hbox {W m}^{-2}$ to the measured upwelling longwave radiation and subsequent energy balance closure largely removed the intercept and at the same time increased $R^2$, as shown in SI Figure 6. Also, the bias between MODIS and plot-scale LST is reduced from − 10.66 K (Table 1) to 4.01 *K* by adding 40 $\hbox {W m}^{-2}$ (approx. 8% of observed $R_{lup}$) and closing the the energy balance.

**Table 1:** Correspondence between daytime landscape-scale LST ($T_{MODIS}$) and plot-scale LST ($T_{s}$) (estimated at TERRA time of pass), using different emissivity.

Sites	Landscape-scale $\varepsilon$			Plot-scale $\varepsilon$ $H = m \Delta T$			Plot-scale $\varepsilon$ $H = m \Delta T +c$			
	$\varepsilon _{land}$	$R^2$	Bias (K)	$\varepsilon _{plot}$	$R^2$	Bias (K)	$\varepsilon _{plot}$	$R^2$	Bias (K)	c ($W m^{-2}$)
SP	0.974	0.81	− 4.61	0.85	0.82	− 1.91	0.92	0.774	− 2.563	18.12
AS	0.974	0.93	− 6.24	0.82	0.93	− 1.92	0.993	0.915	− 4.884	72.46
TT	0.974	0.57	− 8.30	0.80	0.52	− 4.02	0.939	0.521	− 7.466	58.70
HS	0.985	0.16	− 9.90	0.6	0.22	− 2.47	0.949	0.18	− 10.45	237.29
LF	0.985	0.41	− 11.0	0.6	0.41	− 2.57	0.968	0.378	− 11.47	258
AR	0.985	0.27	− 3.51	0.960	0.252	− 2.98	0.996	0.27	− 3.567	14.72
DU	0.985	0.81	4.61	0.985	0.425	− 3.926	0.994	0.405	− 4.603	− 8.11
TUM	0.983	0.84	− 2.10	0.97	0.89	− 1.93	0.955	0.85	− 1.696	− 24.24
BR	0.983	0.937	− 0.195	0.82	0.895	2.72	0.919	0.906	1.662	17.72
YA	0.974	0.855	− 3.45	0.93	0.793	− 0.582	0.873	0.826	0.073	− 22.95

The emissivity values used to retrieve plot-scale LST are either taken from MODIS ($\varepsilon _{land}$), or derived from flux tower data ($\varepsilon _{plot}$), using Eq. (7) ($H = m \Delta T$) or Eq. (8) ($H = m \Delta T + c$). The reported $\varepsilon _{plot}$ and intercept (*c*) are median values over all months for each site. Bias is defined as the mean of $T_{s} - T_{MODIS}$, $R^{2}$ is the coefficient of determination between plot-scale LST and landscape-scale LST. The site acronyms are explained in Table 2.

### Uncertainty in plot-scale $\varepsilon$ and LST

**Figure 6:**
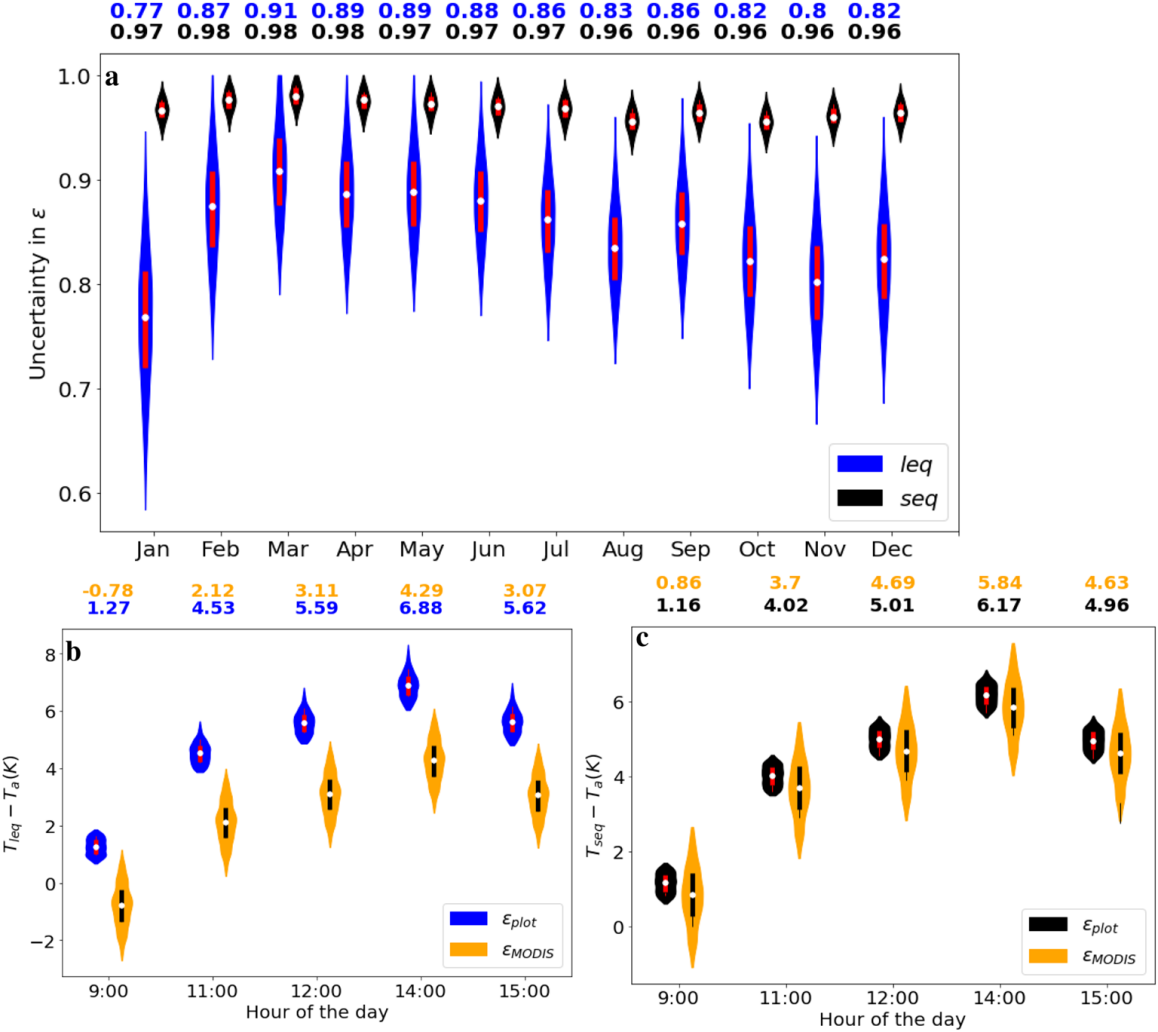
Uncertainty in plot-scale estimations of $\varepsilon$ and surface-air temperature differences ($T_{s} - T_{a}$) at Alice Springs (AS), based on Eq. (7) (no intercept in $H(\Delta T)$). Monthly values of $\varepsilon$ shown for 2017 and hourly $T_{s} - T_{a}$ for 15 August 2017. (**a**) Uncertainty in monthly $\varepsilon _{plot}$ due to uncertainty in *H*, $R_{lup}$, $R_{ldw}$ and $T_{a}$, using Eq. (5) (‘leq’, blue) and Eq. (9) (‘seq’, black). (**b**) Hourly uncertainty in $T_{s} - T_{a}$ on 15 July based on Eq. (5), due to uncertainty in $R_{lup}$, $R_{ldw}$ and $T_{a}$ when landscape-scale emissivity is used ($\varepsilon _{MODIS}$, orange) or due to uncertainty in *H*, $R_{lup}$, $R_{ldw}$ and $T_{a}$ when $\varepsilon _{plot}$ is used (blue). (**c**) Same as Panel b, but based on Eq. (9). The white dots represent the median values of each distribution, the bars extend between the 25 and 75% quantiles and the outer-most violin represent the full distributions of the data.

Each of the observed input variables used for the estimation of plot-scale $\varepsilon$ and LST has an associated uncertainty. Here we present exemplary results for Alice Springs, which showed the highest correlation between plot-scale and landscape-scale LST estimations (Table 1). The uncertainty in plot-scale $\varepsilon$ estimated using Eq. (6) (‘leq’) and Eq. (7) (i.e. without intercept in $H(\Delta T)$) was mainly in the range of ± 0.02 to ± 0.05, with a maximum of ±0.2 if outliers are included (blue color in Fig. 6a). The short equation (Eq. 10, ‘seq’) resulted in a vary narrow range of $\varepsilon$ values between 0.94 and 0.99 throughout the year, with very small uncertainty (around $\pm 0.01$, black boxes in Fig. 6a). Interestingly, the differences in $\varepsilon$ uncertainty did not propagate into differences in LST uncertainty, which were around ±0.2 K at the hourly scale for each equation if plot-scale emissivity was used (blue boxes in Fig. 6b and black boxes in Fig. 6c). In fact, if landscape-scale values of $\varepsilon$ were used, the LST uncertainty was even bigger ($\pm 0.5$ K, orange boxes in Fig. 6b, c). However, if an intercept in the $H(\Delta T)$) relationship was allowed during estimation of $\varepsilon _{plot}$, the uncertainty in $\varepsilon _{plot}$ largely vanished (SI Figure 6a), while the uncertainty in $T_{s} -T_{a}$ at the diurnal scale doubled (SI Figure 6b).

## Discussion

Our analysis revealed a fundamental flaw in the commonly used short equation [Eq. (10)] for estimating plot-scale LST and $\varepsilon _{plot}$, as it does not produce the same results as the long equation [Eq. (6)] even with high values of $\varepsilon _{MODIS}$. In fact, the short equation strongly over-estimates the sensitivity of LST to $\varepsilon$ (SI Figure 4), as it neglects the fact that low emissivity results in a greater fraction of reflected longwave in the sensor signal (compare Eq. (10) and (6)). The sensitivity of the long equation [Eq. (6)] to $\varepsilon$ is driven by the contrast between $R_{lup}$ and $R_{ldwn}$, whereas for the short equation [Eq. (10)], it is only driven by observed $R_{lup}$ (SI Figure 2). For instance, an error of 0.01 in $\varepsilon$ at a water-limited site (e.g. AS) can cause an error of 0.17 K using Eq. (6) and 0.79 K using Eq. (10) respectively (SI Figure 4). This means that small errors in $\varepsilon$ can result in large differences in LST when using the short equation, or conversely, unrealistic LST values can conveniently be rectified by slightly changing the $\varepsilon$ value. This is illustrated e.g. in Fig. 6, where estimation of $\varepsilon _{plot}$ resulted in similar LST values between the short and long equations, but with vastly different $\varepsilon$ values and much greater uncertainty in estimated $\varepsilon$ using the long equation compared to the short equation. Considering that the short equation ignores an important component of longwave radiation, it must be concluded that in this case, it achieves seemingly the right results for the wrong reasons. The reduced sensitivity of the long equation [Eq. (6)] to $\varepsilon$ is of advantage for plot-scale LST estimation, since $\varepsilon _{plot}$ is usually unknown and therefore used as an approximate value^9^. However, when using the long equation in conjunction with plot-scale *H* measurements to estimate $\varepsilon _{plot}$, we obtained unrealistically low $\varepsilon$ values at some sites (e.g. HS and LF, Table 1) in comparison to previously reported $\varepsilon$ values for a soil-vegetation system^28,29^. This strong bias in plot-scale $\varepsilon$ estimates was largely removed if the $H(\Delta T)$ linear fit was allowed to have an intercept (Table 1, plot-scale $\varepsilon$). The intercept (i.e. $\Delta T \ne 0$ at $H=0$) could be caused by combining measurements coming from instruments (radiometer, eddy covariance system) with different footprints^21^. The mismatch of source areas becomes important if the surface underlying the instruments has a heterogeneous land cover. Although “footprint awareness” is often omitted at ECS under the assumption of homogeneity^20^, in patchy vegetation, the radiometer can be “seeing” a different vegetation fraction than that contributing to EC measurements, meaning that $H\not = 0$ at $\Delta T=0$. This problem was not detected by Holmes et al.^10^, as the short equation (Eq. (10)) was used, and due to its high sensitivity to $\varepsilon$ (SI Figure 4(a)) even a small reduction in $\varepsilon$ corrected the offset in $H(\Delta T)$ (Fig. 1a). In contrast, when repeating the same analysis using the long equation [Eq. (6)], a larger reduction in $\varepsilon$ is required to remove the intercept, resulting in lower $\varepsilon$ (Fig. 1b). By allowing an intercept in the $H(\Delta T)$ linear fit, we implicitly account for the possibility of a footprint mismatch or instrument bias in the data. This small change in methodology enables us to detect such problems by inspecting the value of the intercept (*c*). Considering the aerodynamic footprint to be larger than the radiometric footprint^20,21^, a positive intercept can be interpreted as the *H* from the aerodynamic footprint which is not seen by the radiometer.

The intercept was very high for the sites HS and LF (Table. 1). A close inspection of the $H(\Delta T)$ plots at these sites (SI Figure 1) revealed negative day-time $T_{s} - T_{a}$ (Fig. 2), which may suggest an underestimation of $R_{lup}$. While testing this hypothesis at HS (having the highest intercept, Fig. 4c) we found that adding roughly 40 $\hbox {W m}^{-2}$ (approx. 8% of observed $R_{lup}$, Fig. 5) in observed $R_{lup}$ led to significant reduction in the intercept from 294 $\hbox {W m}^{-2}$ (Fig. 4c) to 17 $\hbox {W m}^{-2}$ and positive day-time $T_{s} - T_{a}$ (Fig. 5a). The other linear regression parameters (m, $R^{2}$, RMSE) were not affected (compare Figs. 5a and 4c). The hemispherical view of the radiometers looking down at a heterogeneous canopy makes it possible that they “see” more tree crowns and less soil than the area contributing to the eddy covariance footprint. This could lead to an underestimation of $R_{lup}$, and an underestimation by 30–40 $\hbox {W m}^{-2}$ would be equivalent to approximately 5–10$\%$ of the observed flux, which is within the range of a typical energy imbalance found at this site. Previous studies have found a dependence of footprint mismatch on wind direction^20–22^, but we did not find a significant relation between monthly intercept and dominant wind direction at Howard Springs.

Surface heterogeneity has also been recognized as one of the potential causes for the lack of energy balance closure observed at most ECS at diurnal scales^30,31^. However, in our analysis the use of an energy balance closure scheme (based on the Bowen ratio) led to much lower values of $\varepsilon _{plot}$ using Holmes' approach with the long equation and without intercept. In contrast, if an intercept was allowed, energy balance closure led to an increase in positive intercept (Fig. 5b). Perhaps this is the reason why other studies on plot-scale $\varepsilon$ estimation have also used the observed fluxes without correction^10,11,19^. Other energy balance closure schemes add the missing energy to H in water limited ecosystems^32^, or to LE in energy limited ecosystems^33^. However, our analysis suggests that the footprint mismatch may cause a small bias in the upwelling longwave radiation measurements that is not accounted for in any conventional energy balance closure approaches. When we added 35 $\hbox {W m}^{-2}$ (instead of 40 $\hbox {W m}^{-2}$, see Fig. 5a) to the measured upwelling longwave radiation and subsequently closed the energy balance at the HS site (which had the largest $H(\Delta T)$ intercept), we largely removed the intercept and at the same time obtained realistic $\varepsilon$ values and an increased $R^2$ (SI Figure 6). In addition, the bias between MODIS LST and plot-scale LST at HS was reduced by 6.4 K (SI Figure 6b), compared to using upwelling longwave without correction.

When estimating plot-scale LST using $\varepsilon _{MODIS}$ values, we found at many sites with a sparse canopy strongly negative bias in comparison to MODIS LST, which is in agreement with previous studies where the bias for sparse canopies reached up to 12 K^34^. The MODIS overpass can have a large off-Nadir viewing angle, which would lead to an elongated foot-print^35^ and therefore, a different distribution of bare soil and vegetated areas compared to the mostly Nadir viewing angle of the tower-mounted sensor. The difference in footprint and viewing angles between the tower mounted pyrgeometers and MODIS radiometers could also be the reason for bias between the two LST estimates. Plot-scale LST estimates based on plot-scale $\varepsilon$ using a linear $H(\Delta T)$ fit without an intercept largely reduced this bias between plot-scale and MODIS LST (Table 1) and also reduced the uncertainty in diurnal LST (Fig. 6b, c) in comparison to the use of $\varepsilon _{MODIS}$. However, the resulting plot-scale $\varepsilon$ values were unrealistically low at some sites (Table 1, center). In contrast, allowing an intercept ($H=m \Delta T + c$) in $\varepsilon _{plot}$ estimation resulted in more realistic $\varepsilon$ values at these sites, but very large intercept values (over 200 $\hbox {W m}^{-2}$ at some sites), indicating that the plot-scale LST values cannot be used in combination with the observed aerodynamic fluxes at these sites, as strongly positive *H* at 0 surface-air temperature difference is physically inconsistent (Fig. 4c). In addition, this approach increased the bias between plot-scale and MODIS LST at most of the study sites (Table 1). Note that the correspondence between landscape-scale LST and plot-scale LST can vary strongly between sites, depending on canopy densities and viewing angles (tower vs. satellite)^35^, sensor installation height and position, and sensor types^21^. At the sites with the largest intercept values, we found that an assumed bias in upwelling longwave radiation by only 6–9% would largely remove the intercept and also reduce the bias between MODIS and plot-scale LST (Fig. 5a, SI Figure 6b). A detailed analysis of such bias and potential correction approaches is beyond the scope of this study. Given that the fit of a linear model without intercept is statistically questionable in general^36^, and the fact that such a fit resulted in unrealistically low values of $\varepsilon$ at some sites, we conclude that fitting a model with intercept is the more robust approach, and that a significant intercept should be used as a red flag for the utility of the data for estimation of plot-scale LST. Additionally, the uncertainty in $\varepsilon _{plot}$ values obtained using a regression model with intercept nearly vanished in comparison to the uncertainty resulting from a regression model without intercept (see SI Figure 6a).

Note that the fluxes observed at ECS are representative of the composite signal from both, soil and vegetation, which typically have different ranges of surface temperatures and emissivities^37^. The $\varepsilon$ of soil strongly depends on soil moisture content^38^, whereas the $\varepsilon$ of a canopy depends on its structural attributes and leaf area index, the latter of which can vary strongly at the seasonal scale^39^. For example, the laboratory-measured directional $\varepsilon$ for various canopy elements (bark, leaf and its arrangement, stem wood) ranged between 0.9 and 1 at the Yatir site^40^. Laboratory measurements of thermal infrared reflectance spectra suggest that the $\varepsilon$ uncertainty due to structural unknowns, such as leaf orientation, is more significant than the differences in leaf component emissivity among plant species^29^. Consequently, it is clear that the $\varepsilon$ of a surface is a function of many factors and a detailed analysis of all these factors is out of scope of the present study. Derivation of landscape-scale broadband emissivity ($\varepsilon _{MODIS}$) from narrowband spectral emissivity is a first-order approximation for capturing the integrated effects of land cover from MODIS spectral bands^37^, whereas the derivation of $\varepsilon _{plot}$ from EC flux data provides an independent alternative for the estimation of effective plot-scale $\varepsilon$. Our finding that inclusion of an intercept in the $H(\Delta T)$ relationship when estimating $\varepsilon _{plot}$ significantly reduces uncertainty in $\varepsilon _{plot}$ while increasing uncertainty in $\Delta T$ suggests that this method could be used for reliable estimates of effective $\varepsilon _{plot}$ within the radiometer footprint even in the presence of a footprint mismatch between the radiometric and *H* measurements. The approach could also be extended to urban settings if reliable eddy covariance measurements are available and anthropogenic heat components are known. Although the effects of footprint mismatch between radiometric and eddy covariance measurements could be large in such a heterogeneous setting, $\varepsilon _{plot}$ estimation based on $H(\Delta T)$ with intercept could provide a robust estimate of effective $\varepsilon _{plot}$, which is important for climate models simulating urban heat island effects^41^.

In summary, our results reveal that the short equation (Eq. (10), neglecting downwelling longwave radiation) leads to biased estimates of LST and substantially over-estimated sensitivity of LST to surface emissivity. Therefore, the use of Eq. (10) is not recommended and should be replaced by Eq. (6) if downwelling longwave radiation measurements are available. At some sites, the use of Eq. (6) resulted in plot-scale LST estimates that were far below satellite-derived landscape-scale LST values, and also inconsistent with plot-scale flux data (negative surface-air temperature difference when sensible heat flux is strongly positive). In many previous studies, such bias would have been removed by slightly lowering surface emissivity ($\varepsilon$), but the reduced sensitivity of Eq. (6) to $\varepsilon$ would require unrealistically low values of $\varepsilon$ to remove the low-bias in LST. When estimating plot-scale $\varepsilon$ values, realistic estimates based on Eq. (6) are only possible at these sites if we include an intercept in the $H (\Delta T)$ relationship, but this again results in very high intercept values (over 200 W $\hbox {m}^{-2}$). Note that high values of intercept do not necessarily make $\varepsilon _{plot}$ unreliable, they rather suggest poor correspondence between *H* and $T_{s}$ due to footprint mismatch. Hypothesizing that the intercept is a consequence of a footprint mismatch between the aerodynamic and radiometric measurements, a small correction in upwelling longwave (6–9%) and subsequent energy balance closure (based on the Bowen ratio) largely removed the intercept and produced realistic $\varepsilon _{plot}$ values and self-consistent $H(\Delta T)$ plots. This approach also reduced the bias between plot-scale LST and MODIS LST, although it did not improve the weak correlation between these LST estimates (SI Figure 5(b)). In the past, ground-based radiometric measurements have been used for the validation of the MODIS LST product^42^, therefore our study compared the new plot-scale LST estimates with MODIS LST to check their correspondence.

The combination of radiometric and aerodynamic measurements for the estimation of $\varepsilon _{plot}$ and LST provides a quality check on the correspondence between observed fluxes and temperatures at ECS. The intercept value can be used as a consistency criterion for observed data (radiometric and aerodynamic measurements) before using them in combination, as a strong intercept indicates inconsistency between observed sensible heat flux and surface-to-air temperature difference. Therefore, the proposed method of fitting a linear relation with intercept to *H* and $\Delta T$ has the potential to provide more reliable benchmark data sets for model evaluation and validation at the ecosystem scale (plot-scale). The $\varepsilon _{plot}$ estimates could also be used to parameterize climate and weather prediction models at ecosystem scale, but this was not tested in the present study. Overall, the implications of our study are of particular relevance for the research community interested in process-based understanding of the diurnal and seasonal feedbacks in soil-vegetation systems based on observed fluxes.

## Methods

In the last two decades, plot-scale radiometric data collected at ECS have gained popularity for in-situ LST retrieval due to its high temporal resolution^31,43^. In addition to this, the LST estimates at plot-scale originate from a relatively homogeneous footprint in comparison to the satellite-derived LST (MODIS pixels). This section describes: (i) how to retrieve plot-scale LST and $\varepsilon$ using eddy covariance measurements, (ii) how to quantify the correspondence between plot-scale LST with MODIS LST and, (iii) how to quantify the uncertainty in plot-scale LST and $\varepsilon$.

### Tower data

ECS collect micro-meteorological measurements above the surface (vegetation canopy) using towers (flux tower) following common measurement protocols^44^. The towers are generally equipped with pyrgeometers or radiometers to measure upwelling and downwelling shortwave and longwave radiation, which is further used to calculate net radiation (Eq. 2). Besides radiative fluxes, measurement at ECS also include sensible and latent heat fluxes, net carbon-dioxide exchange and a range of meteorological variables, such as air temperature ($T_{a}$), humidity and wind speed. $T_{a}$ is the air temperature measured at a reference height above the canopy. Each flux measurement is accompanied by a flagging system based on the second CarboEurope-IP QA/QC workshop^45^. In our current work, we use high quality available data (flag 0) as it is without atmospheric corrections. For the analysis, ten sites were selected to represent a variety of land cover types and climates (Table 2). Eight sites belong to the North Australian Tropical Transect (NATT) and two sites (Yatir Forest, Brookings) are chosen to replicate results from Holmes et al.^10^ as shown in Table 2. Eddy covariance level 3 data is obtained from http://data.ozflux.org.au/portal/pub/listPubCollections.jspx for Australian sites. The data for Brookings was obtained from Ameriflux whereas the data for Yatir Forest was obtained through personal communication with Professor Yakir’s lab in order to obtain the older version of the data, which was used by Holmes et al.^10^.

### MODIS data

Landscape-scale emissivity and LST data (MODIS product MOD11A1) was downloaded from NASA earth data https://lpdaac.usgs.gov/. It is a level 3 daily LST product gridded in the sinusoidal projection at a spatial resolution of 0.928 km by 0.928 km. The daily LST pixel values in each granule (tile contains 1200 x 1200 grids in 1200 rows and 1200 columns) is retrieved by the generalized split-window algorithm under clear-sky conditions and MODIS LST values are averaged by overlapping pixels in each grid with overlapping areas as weight^46^. The downloaded data in hierarchical data format (hdf), were converted into tagged image file format (tiff) using the python package PyModis^47^. Alternatively MODIS data can also be obtained from https://appeears.earthdatacloud.nasa.gov/. MODIS measures spectral emissivity through four channels (28, 29, 30, 31) at wavelengths ranging between 8 and 12 $\upmu \hbox {m}$^37^ and the system of equations is iteratively solved for a given range of wavelengths (8–12 $\upmu \hbox {m}$) to obtain $\varepsilon$ and LST using radiative transfer models^23,37,48^. In the current study, dataset columns used to compare plot-scale LST are day time daily LST and local view time. In order to obtain landscape-scale $\varepsilon$, the emissivity from bands 31 and 32 are used. These bands have stable emissivities than other channels ranging from  0.92 to 1, and can be used to derive broadband emissivity^46^.

**Table 2:** Description of study sites.

Study site	Latitute, longitude	Landcover	Time-period	Longwave sensors	Sensor installation height (m)	Altitude (m)
Sturt Plains (SP)	$-$ 17.1507, 133.3502	Mitchell Grass	2016–2019	pyrgeometers (CG-2)	4.8	230
Alice Springs (AS)	$-$ 22.2828, 133.2493	Mulga woodland, hummock grassland, river red gum forest	2016–2018	Radiometer (CNR1)	12.2	606
Ti Tree East (TT)	$-$ 22.2870, 133.6400	Grassy mulga woodland, Corymbia/Triodia savanna	2016–2018	Radiometer (CNR1)	9.9	553
Howard Springs (HS)	$-$ 12.4943, 131.1523	Open woodland savanna	2016–2018	Pyrgeometers (CM-7B, CG-2)	23	63
Litchfield (LF)	$-$ 13.1790, 130.7945	Tropical savanna	2016–2018	Radiometer (CNR4)	31	222
Adelaide River (AR)	$-$ 13.0769, 131.1178	Savanna dominated by Eucalyptus tectifica and Planchonia careya	2006–2009	Pyrgeometers (CNR1)	15	90
Daly Uncleared (DU)	$-$ 14.1592, 131.3881	Woodland savanna	2016–2018	Radiometer (NRlite)	21	110
Tumbarumba (TUM)	$-$ 35.6566, 148.1517	Wet sclerophyll	2015–2018	Pyrgeometers (CM3 and CG3)	70	1200
Brookings (BR)	44.352, 96.840	Cropland	2005	Pyrgeometers^49^	NA	510^42^
Yatir Forest (YF)	31.344894, 35.051922	Evergreen needleleaf forest	2005	Pyrgeometers^49^	NA	641

### Plot-scale $\varepsilon$ and LST estimation

**Figure 7:**
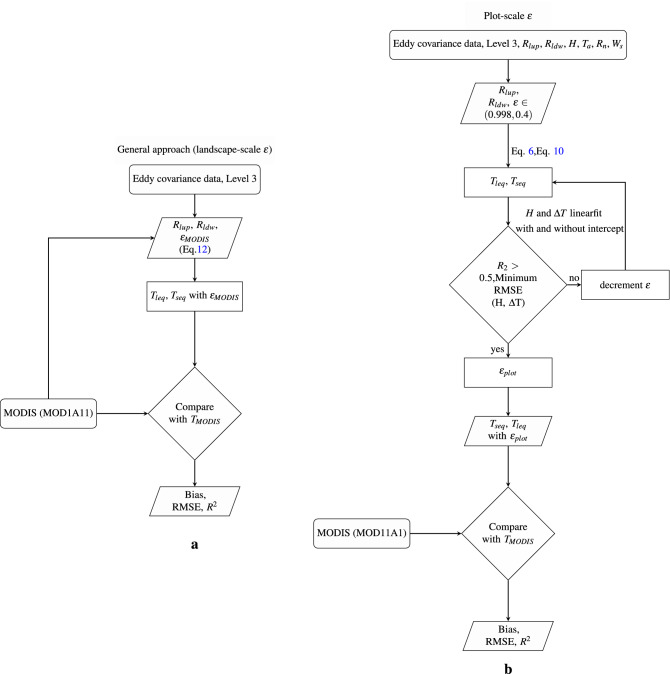
Schematic representation of steps followed for plot-scale LST retrieval using landscape-scale emissivity (**a**) or plot-scale emissivity (**b**). To estimate plot-scale $\varepsilon _{plot}$, surface-air temperature difference ($\Delta T$) is computed from observed longwave radiation ($R_{lup}$ and $R_{ldwn}$) and $T_{a}$ for given $\varepsilon _{plot}$, and then $\varepsilon _{plot}$ is varied in a way to minimise RMSE of a linear relationship between observed sensible heat flux (H) and $\Delta T$. The resulting surface temperatures ($T_{leq}, T_{seq}$) are then compared to $T_{MODIS}$, with the $R^2$, RMSE, and bias reported in Fig. (3).

LST is defined as the “ensemble directional radiometric surface temperature”^18^, and can be estimated from the infrared radiance emanating from a given surface with known emissivity^50^. The emissivity at ecosystem-scale can also be estimated using observed *H*, $R_{lup}$, $R_{ldwn}$, and $T_{a}$. A plot-scale $\varepsilon$ ($\varepsilon _{plot}$) estimation approach was initially proposed by Holmes^10^ using the short equation (Eq. 10). In the present work, we have used both the long equation (Eq. 6) and the short equation (Eq. 10) to estimate $\varepsilon _{plot}$. The prime variables used in the study are *H*, $R_{lup}$, $R_{ldwn}$, and $T_{a}$, whereas the ancillary variables $R_{n}$ and wind speed ($W_{s}$) are used to filter the data for analysis. The data filtering criteria are sufficient net radiation ($R_{n} > 25$ $\hbox {W m}^{-2}$) and wind speed ($W_{s} > 2\,\hbox {m s}^{-1}$)^10^. For each month, a linear regression (with and without intercept, see main text) between sensible heat (*H*) and $T_{s}-T_{a}$) is performed (Fig. 7b) using Scipy (https://docs.scipy.org/doc/scipy/reference/generated/scipy.stats.linregress.html). $T_{s}$ is estimated by solving Eq. (6) and Eq. (10) using measured longwave radiation and prescribed $\varepsilon$, starting with the maximum possible value for a grey body, 0.99, and then progressively reducing $\varepsilon$ with step size of 0.002 until we reach a minimum RMSE for a linear relationship between *H* and $\Delta T$. Only months with $R^{2}>0.5$ between *H* and $\Delta T$ are considered for $\varepsilon _{plot}$ estimation. An illustration plot for RMSE as a function of $\varepsilon$ is shown in SI Figure 5. The monthly $\varepsilon _{plot}$ is obtained using the long [Eq. (6)] and short equation [Eq. (10)] and termed as $\varepsilon _{leq}$ and $\varepsilon _{seq}$ respectively, as shown in Fig. 7b. For two sites with a high value of intercept (HS and LF in Table 1) we tested if adding 6–8% to the observed $R_{lup}$ and closing the energy balance using Bowen ratio closure before $\varepsilon _{plot}$ estimation would remove the intercept (Fig. 5).

Recently, another approach for plot-scale $\varepsilon$ estimation using Eq. (4) was used by Maes et al.^11^. In this approach, data sets are filtered for non rainy days without snow cover ($\alpha < 0.4$) and near-zero *H* ($-2< H <2$). The $\varepsilon$ values are then estimated by substituting $T_{s} = T_{a}$ in Eq. (4) as shown in Eq. (11). The monthly $\varepsilon$ was obtained as the median of $\varepsilon$ obtained by substituting filtered data in Eq. (11) (red stars in Fig. 1).

11 $$\begin{aligned} \varepsilon = \frac{R_{ldwn} - R_{lup}}{R_{ldwn} - T_{a}^{4} \sigma } \end{aligned}$$

### LST comparison

MODIS LSTs are a global reference for LST and used world-wide, also in conjuction with plot-scale flux measurements. To calculate plot-scale LST for the exact time of TERRA day-time overpass for each site, the 30 minute tower data was interpolated linearly, and the interpolated $R_{ldwn}$ and $R_{lup}$ observations corresponding to the time of overpass were used in conjunction with the monthly $\varepsilon _{plot}$ or $\varepsilon _{MODIS}$ for the calculation. Plot-scale daily LST is compared to MODIS LST in terms of the mean, bias, RMSE and $R^2$ using a robust linear regression model (scipy stat model) as shown in Fig. 7a. The goodness of fit between plot-scale and landscape-scale LST was determined by looking at $R^2$ (Fig. 7b). The bias is estimated as the mean of the deviation between daily MODIS LST and plot-scale $T_{s}$. See SI Table 1 for data sources and acronyms.

### General approach

We estimate landscape-scale broadband $\varepsilon$ using MODIS spectral $\varepsilon$ as shown in Bahir et al.^51^.

12 $$\begin{aligned} \varepsilon _{MODIS}= 0.4587 \varepsilon _{31} + 0.5414 \varepsilon _{32} \end{aligned}$$

Tower-based longwave radiation measurement ($R_{lup}$, $R_{ldwn}$) passing the filtering criteria (as mentioned in plot-scale emissivity estimation) along with MODIS based $\varepsilon$ was used to invert LST using Eqs. (6) and (10). The obtained plot-scale LST was compared to landscape-scale MODIS LST using a robust linear regression as mentioned above and shown in Fig. 7a.

### Uncertainty estimation

Uncertainty in plot-scale $\varepsilon$ and LST was quantified based on an assumed systematic error (caused by a potential bias in measurement devices) at the study sites. In a first step, based on the literature^24,52^, the error bounds of each input variable (*H*, $R_{lup}$, $R_{ldw}$, $T_{a}$) used for plot-scale $\varepsilon$ estimation were defined. The error bounds for $R_{lup}$ and $R_{ldwn}$ are − 5 to 5 $\hbox {W m}^{-2}$^24^, for *H*, we used − 20 to 20 $\hbox {W m}^{-2}$ and for $T_{a}$ we used − 1 to 1 K^52^. The error samples (perturbation) within these bounds were generated using the Saltelli sampling scheme (using the python package SALIB^53^). Each error sample is added to the monthly segregated measured fluxes as explained above. Observed fluxes combined with perturbed fluxes are used to estimate $T_{s}$ using Eqs. (10) and (6). The obtained range of diurnal $T_{s}$ and observed $T_{a}$ based on the perturbation is used to calculate the uncertainty in $\Delta T$; an example for July 15 is shown in Fig. 6c. Perturbed sensible heat flux ($H +$sample error) and perturbed $\Delta T$ is used to obtain $\varepsilon _{plot}$ as described above. The distribution of monthly $\varepsilon _{plot}$ values is reported as uncertainty in monthly $\varepsilon$.

## Supplementary Information

Supplementary Information.

## Data Availability

The data and code used for this study is freely available from zenodo.org (https://doi.org/10.5281/zenodo.6385016).
