# Supplementary material for: Downwelling longwave radiation and sensible heat flux observations are critical for surface temperature and emissivity estimation from flux tower data
Source: Sci Rep. 2022 May 21;12:8592. doi: 10.1038/s41598-022-12304-3 (PMC9124221; doi:10.1038/s41598-022-12304-3)
Supplement: Supplementary file 1 — Supplementary Information. [file 41598_2022_12304_MOESM1_ESM.pdf]

Downwelling longwave radiation and sensible heat flux observations are critical for surface temperature and emissivity estimation from flux tower data

Gitanjali Thakur[1,*], Stanislaus J. Schymanski[1,*], Kaniska Mallick [1], Ivonne Trebs[1], and Mauro Sulis[1]

[1]Luxembourg Institute of Science and Technology, ERIN, Belvaux, L-4422, Luxembourg
[*]gitanjali.thakur@list.lu
[*]stanislaus.schymanski@list.lu

ABSTRACT

Land surface temperature (LST) is a preeminent state variable that controls the energy and water exchange between the Earth's surface and the atmosphere. At the landscape-scale, LST is derived from thermal infrared radiance measured using space-borne radiometers. In contrast, plot-scale LST estimation at flux tower sites is commonly based on the inversion of upwelling longwave radiation captured by tower-mounted radiometers, whereas the role of the downwelling longwave radiation component is often ignored. We found that ignoring the reflected downwelling longwave radiation leads not only to substantial bias in plot-scale LST estimation, but also have important implications for the estimation of surface emissivity on which LST is co-dependent. The present study proposes a novel method for simultaneous estimation of LST and emissivity at the plot-scale and addresses in detail the consequences of omitting down-welling longwave radiation as frequently done in the literature. Our analysis uses ten eddy covariance sites with different land cover types and found that the LST values obtained using both upwelling and downwelling longwave radiation components are 0.5 to 1.5 K lower than estimates using only upwelling longwave radiation. Furthermore, the proposed method helps identify inconsistencies between plot-scale radiometric and aerodynamic measurements, likely due to footprint mismatch between measurement approaches. We also found that such inconsistencies can be removed by slight corrections to the upwelling longwave component and subsequent energy balance closure, resulting in realistic estimates of surface emissivity and consistent relationships between energy fluxes and surface-air temperature differences. The correspondence between plot-scale LST and landscape-scale LST depends on site-specific characteristics, such as canopy density, sensor locations and viewing angles. Here we also quantify the uncertainty in plot-scale LST estimates due to uncertainty in tower-based measurements using the different methods. The results of this work have significant implications for the combined use of aerodynamic and radiometric measurements to understand the interactions and feedbacks between LST and surface-atmosphere exchange processes.

Supplementry Information

SI1. Abbreviation list

Symbol	Description	Unit
R_{net}	Net radiation	W m^{-2}
H	Sensible heat flux	W m^{-2}
LE	Latent heat flux	W m^{-2}
G	Ground heat flux	W m^{-2}
R_{lem}	Emitted longwave radiation	W m^{-2}
ε	Surface emissivity	(-)
σ	Stefan-Boltzmann constant	W $m^{-2}K^{-4}$
T_s	Surface temperature	K
R_{sdwn}	Down-welling shortwave	W m^{-2}
R_{ldwn}	Down-welling longwave	W m^{-2}
R_{sref}	Reflected shortwave	W m^{-2}
α	Surface albedo	(-)
m	Aerodynamic conductance to heat transport	(m/s)
ε 31	Spectral emissivity for wavelength of 11 μm	(-)
ε 32	Spectral emissivity for wavelength of 12 μm	(-)
BADAM	Ameriflux dataset	(-)
TERRA	NASA scientific research satellite	(-)
NATT	North Australian Tropical Transect	(-)

SI Table 1. Abbreviation list

SI2. Comparison table of plot-scale LST with landscape LST using landscape and plot-scale ε

Sites	Landscape-scale ε					Plot-scale ε					
	ε	seq		leq		seq			leq		
		R^2	bias	R^2	bias	opt ε	R^2	bias	opt ε	R^2	bias
SP	0.974	0.80	-3.67	0.81	-4.61	0.96	0.81	-3.0	0.85	0.82	-1.91
AS	0.974	0.93	-4.78	0.93	-6.31	0.96	0.93	-3.4	0.82	0.93	-1.92
TT	0.974	0.55	-6.76	0.57	-8.30	0.95	0.58	-5.06	0.80	0.52	-4.02
HS	0.985	0.16	-8.89	0.16	-9.90	0.92	0.21	-4.78	0.6	0.22	-2.47
LF	0.985	0.40	-10.0	0.41	-11.0	0.92	0.40	-4.41	0.6	0.41	-2.57
AR	0.985	0.18	-2.61	0.27	-3.51	0.997	0.23	-2.93	0.96	0.252	-2.98
DU	0.985	0.80	-3.67	0.81	-4.61	0.99	0.428	-3.682	0.985	0.425	-3.926
TUM	0.983	0.82	-2.27	0.84	-2.10	0.99	0.89	0.99	0.97	0.89	1.93
BR	0.983	0.937	0.525	0.937	-0.195	0.98	0.917	1.87	0.82	0.895	2.72
YA	0.974	0.855	-2.081	0.855	-3.45	0.97	0.522	-4.517	0.93	0.793	-0.582

SI Table 2. Comparison of plot-scale LST with landscape-scale daytime LST (MODIS, MODA001) at TERRA daily time of pass. Plot scale LST is obtained using landscape-scale emissivity (MODIS ε) (left column) and plot-scale emissivity obtained considering no intercept in H and ΔT (Optimum ε) at study sites. The reported plot-scale emissivity are median values and landscape emissivity are using channel 31 and 32 of MODA001 dataset. Bias is defined as mean of $T_s - T_{MODIS}$, R^2 is coefficient of determination between plot-scale LST in comparison to landscape-scale LST. The site acronyms can be found in **Table 2** of the main paper.

SI3. Emissivity estimation at Howard Springs and negative $T_s - T_a$

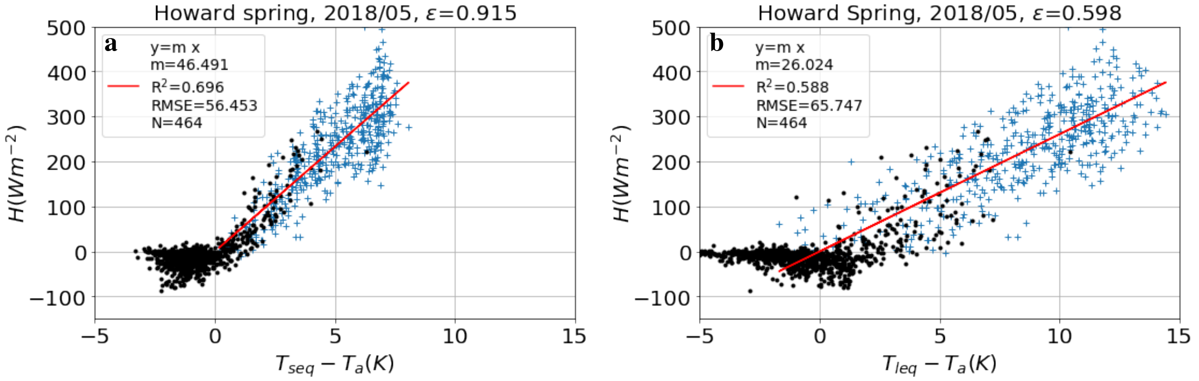

SI Figure 1. Monthly daytime ($R_n > 25 W m^{-2}$) H and ΔT regression plots at Howard Springs using short and long equation. The value of optimised emissivity along with the year and month are shown on top of the plot.

SI4. T_s sensitivity to emissivity at Alice spring and Tumbarumba

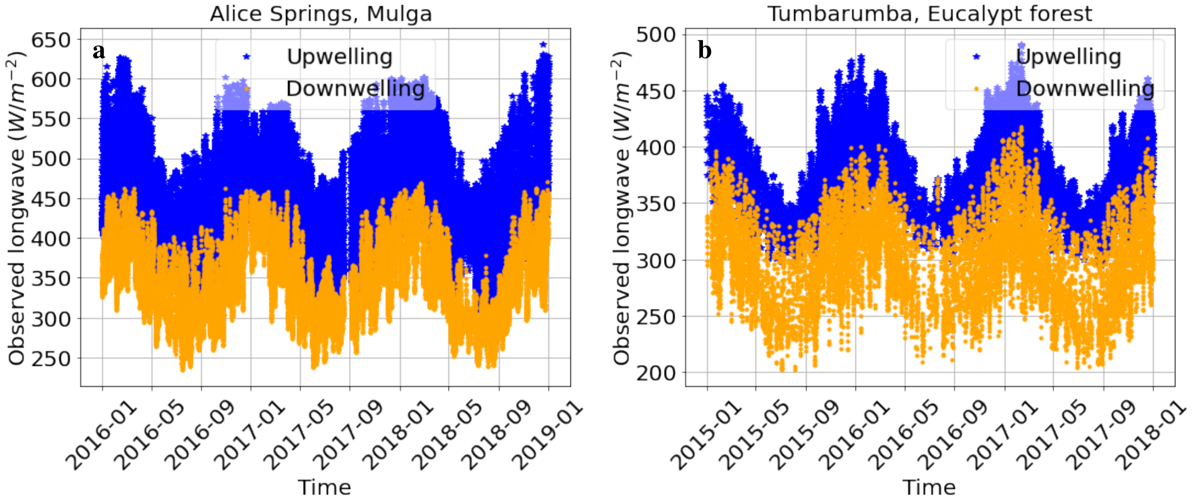

SI Figure 2. Timeseries of up-welling and down-welling longwave at sites having different land cover. (a) Alice Springs a mulga site. (b) Tumbarumba wet eucalypt forest

SI5. Variation of RMSE for monthly of $H(\Delta T)$ plot with surface emissivity

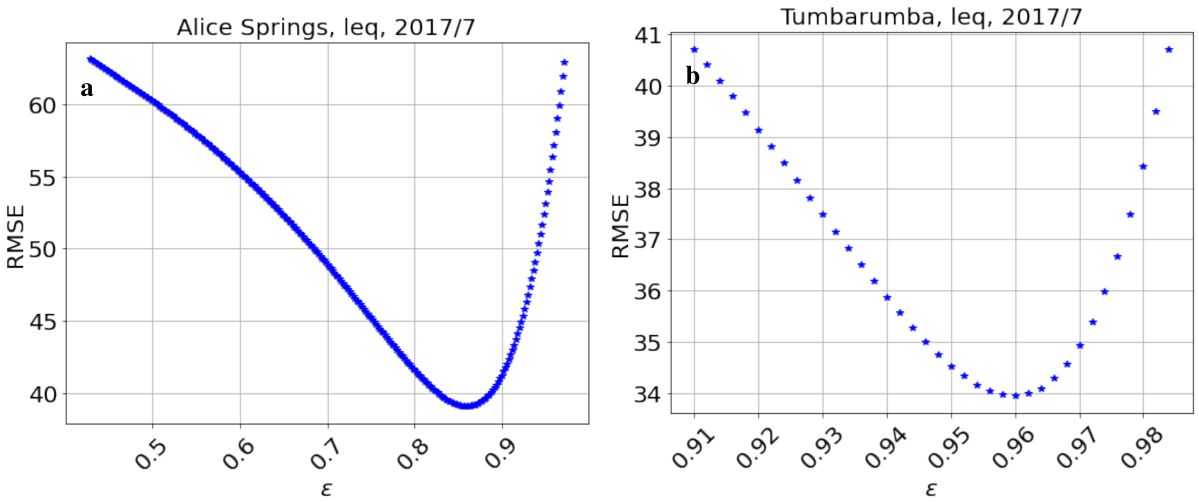

SI Figure 3. The RMSE and emissivity for $H(\Delta T)$ linear fit using long equation (a) Alice Spring (b) Tumbarumba

SI6. Sensitivity of short and long equation to surface emissivity

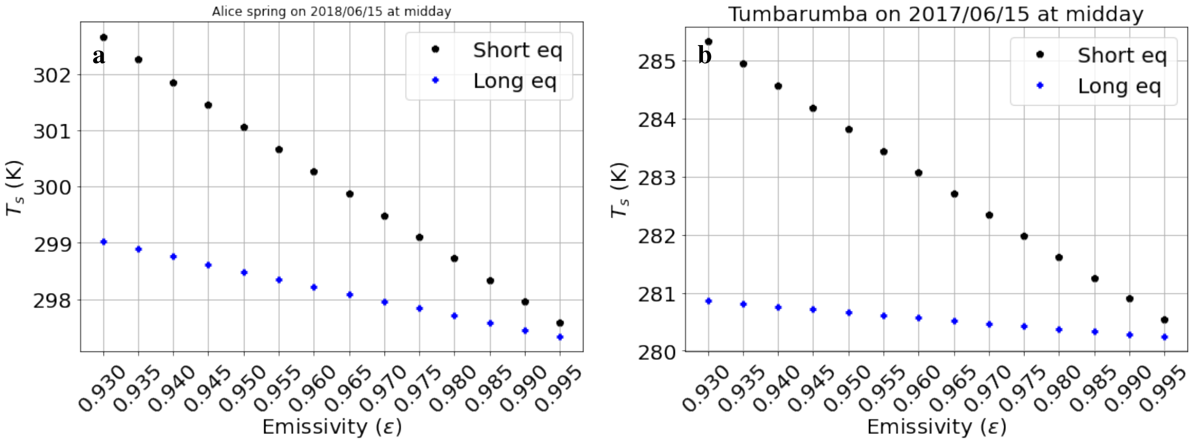

SI Figure 4. Sensitivity of LST estimated using two equations to the range of Broadband emissivity The black dots and blue Stars depicts LST using short (**Eq. 7** of main paper) and long (**Eq. 12** of main paper). Midday longwave measurement for 15June, 2016 at Alice Springs and Tumbarumba is used

SI7. Energy imbalance closure reduces the correction in measured the up-welling longwave at Howard Springs

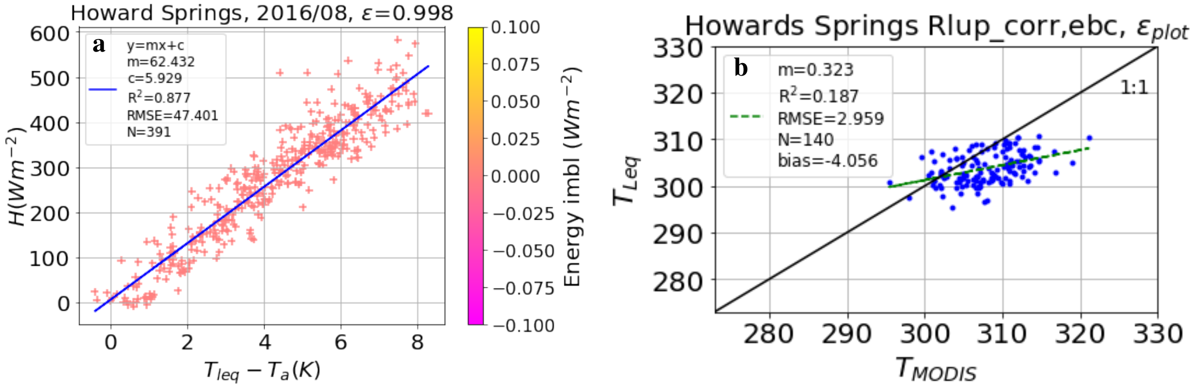

SI Figure 5. (a) Sensible heat flux as a function of surface-to-air temperature difference based on **Eq.10** of the main paper. Same analysis and legends as in **Fig 4c** of main paper, but after adding 35 (Wm^{-2}) to measured R_{lup} and closing the energy balance using a Bowen ratio closure scheme. (b) Comparison of surface temperatures from (a) with landscape scale LST from MODIS.

SI8. SOBOL based uncertainity in epsilon and LST using long equation with accepting intercept in $H(\Delta T)$ and without intercept in $H(\Delta T)$

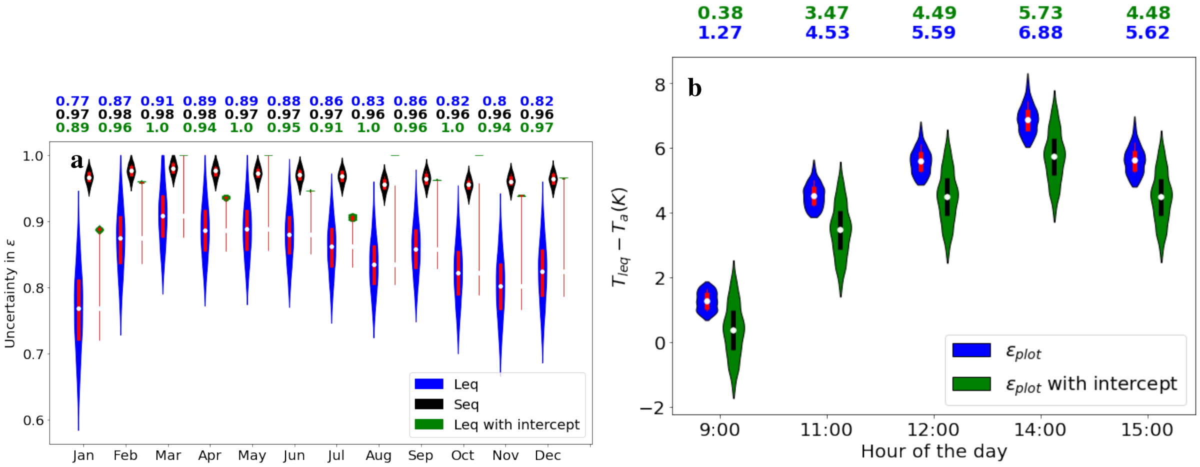

SI Figure 6. (a) Uncertainty in plot-scale ε using the short equation and long equation (with and without intercept in H and ΔT plots). (b) Uncertainty in hourly ΔT using long equation with and without intercept for July 15.
